# Fused in Sarcoma: Properties, Self-Assembly and Correlation with Neurodegenerative Diseases

**DOI:** 10.3390/molecules24081622

**Published:** 2019-04-24

**Authors:** Chen Chen, Xiufang Ding, Nimrah Akram, Song Xue, Shi-Zhong Luo

**Affiliations:** Beijing Key Laboratory of Bioprocess, College of Life Science and Technology, Beijing University of Chemical Technology, Beijing 100029, China; 2017201117@mail.buct.edu.cn (C.C.); 13552909516@163.com (X.D.); nimrahchaudhry@outlook.com (N.A.)

**Keywords:** fused in sarcoma (FUS), prion-like domains, self-assemble, phase separation, phase transition, aggregation, neurodegenerative diseases

## Abstract

Fused in sarcoma (FUS) is a DNA/RNA binding protein that is involved in RNA metabolism and DNA repair. Numerous reports have demonstrated by pathological and genetic analysis that FUS is associated with a variety of neurodegenerative diseases, including amyotrophic lateral sclerosis (ALS), frontotemporal lobar degeneration (FTLD), and polyglutamine diseases. Traditionally, the fibrillar aggregation of FUS was considered to be the cause of those diseases, especially via its prion-like domains (PrLDs), which are rich in glutamine and asparagine residues. Lately, a nonfibrillar self-assembling phenomenon, liquid–liquid phase separation (LLPS), was observed in FUS, and studies of its functions, mechanism, and mutual transformation with pathogenic amyloid have been emerging. This review summarizes recent studies on FUS self-assembling, including both aggregation and LLPS as well as their relationship with the pathology of ALS, FTLD, and other neurodegenerative diseases.

## 1. Introduction

Fused in sarcoma (FUS) is a DNA/RNA binding protein containing 526 amino acids. The *FUS* gene was initially identified as a fusion oncogene on chromosome 16 in human liposarcoma [1], the translocation and fusion of which to transcription factors results in strong transcriptional activation of the proteins. FUS is one of the components of the heterogeneous nuclear ribonucleoprotein (hnRNP) complex. Increasing evidence suggests that FUS is involved in various cellular processes, including gene transcription and regulation, DNA repair, RNA shearing, RNA transport, translation, processing of microRNAs, and maintenance of genomic stability [2]. FUS can bind to RNA, ssDNA, and possibly to dsDNA [3]. The most important binding sequence of FUS is GUGGU, which is rich in 5′ untranslated regions (UTRs), and mutant FUS preferentially binds 3′-UTR and intron sequences [4,5,6]. As an abundant nuclear protein, FUS forms stable complexes with numerous members of the hnRNP family, and some components of the FUS–hnRNP complex have RNA-binding activity [7,8]. The physiological functions of FUS are apparently diverse. Here, we describe its role in RNA metabolism, DNA repair, and its relationship to disease.

### 1.1. Role of FUS in RNA Metabolism

FUS is well-studied for its transcriptional regulation function in cells. FUS binds to single-stranded DNA motifs in the promoter region of certain genes, which are transcribed by RNA polymerases II and III, and then accumulates near the transcription start site (TSS), resulting in changes of transcription levels [9,10]. Additionally, FUS also regulates transcription levels partly via the interaction with specific transcription factors (e.g., Spi-1/PU.1, NF-κB, and Runx2) and transcription initiation factor TFIID [11,12,13,14]. One recognized mechanism of this regulation is that FUS binds to and recruits RNA polymerase II, subsequently altering its phosphorylation status [15]. RNA can promote the oligomerization of FUS, allowing it to form high-order components near TSS, which interact with CTD of RNA polymerase II. This allows more RNA polymerase II to be recruited to TSS and also prevents premature phosphorylation of Ser2 in CTD, stimulating the transition of polymerase from initiation to activity extension [5]. FUS also interacts with the DNA binding domain of certain nuclear hormone receptors to initiate their transcription [16]. Immunofluorescence studies have found that FUS preferentially localizes on active chromatin to execute its role in transcription regulation [17]. Specifically, during meiosis, FUS binds to autosomes other than transcriptionally silent sex chromosomes [18]. Similarly, it does not bind to transcriptionally silenced chromatin during mitosis [19].

FUS modulates RNA splicing in conjunction with splicing regulators or precursor mRNAs [19]. FUS is a transporter of mRNA between the cytoplasm and the nucleus. For instance, the transfer of mRNA between neuronal dendrites and dendritic spines by FUS is essential for neuronal cell maturation, plasticity, and dendrite integrity [20]. Other relevant studies have indicated that FUS boosts the synthesis of microRNAs, as FUS is an integral part of the Drasha complex, which is required as a ribonuclease III enzyme for microRNA biosynthesis [21].

### 1.2. Role of FUS in DNA Damage Repair

During DNA damage repair, FUS is one of the proteins firstly recruited to the DNA damage site [22,23]. Loss of FUS results in impaired ATM/γH2AX (ataxia telangiectasia-mutated gene) signaling. The mechanism by which FUS regulates DNA damage repair also depends on interactions with histone deacetylase 1 (HADC1) and poly-ADP ribose, a by-product of DNA damage [22,24]. Knockdown of FUS during cell growth leads to defects in DNA damage recovery, and disease-associated mutations may limit FUS participation in DNA damage response (DDR) [25].

In 1999, Baechtold demonstrated that FUS participates in DDR, which promotes complementary ssDNA annealing and single-stranded oligonucleotide uptake of homologous supercoiled DNA to form a D-loop. The D-loop formation is a principal step in DNA double-strand break repair via recombination, and the oncogenic fusion form FUS–CHOP does not promote DNA pairing [26]. Wild-type FUS can be phosphorylated by ATM in response to DNA double-strand breaks (DSBs), as phosphorylated FUS binds to dsDNA cleavage and Holliday junctions [27]. When DNA damage occurs, FUS is recruited by sense and antisense noncoding RNAs transcribed from 5′ regulatory regions of the cyclin D1 (CCND1) gene [28]. FUS directly interacts with the CREB-binding protein/p300 histone acetyltransferase via its N-terminal domain, resulting in inhibition of CCND1 transcription [28]. Interestingly, FUS’s ability to respond to DNA damage relies on allosteric interactions with single-stranded, low-copy-number long noncoding RNA transcripts [28]. Although the detailed processes of FUS in DDR have not been fully illustrated, its important function in DDR is without question.

### 1.3. The Link between FUS and Neurodegenerative Disease

Amyotrophic lateral sclerosis (ALS or Lou Gehrig’s disease) is a well-known neurodegenerative disease caused by the loss of both upper and lower motor neurons, first described by the French neurologist Jean-Martin Charcot in 1869 [29]. The first mutation of *FUS* associated with ALS was discovered in 2009 and later on FUS-immunoreactive cytoplasmic inclusions were found in ALS–FUS patients [30]. Shortly thereafter, FUS aggregation has been observed in various neurodegenerative diseases, such as frontotemporal lobar degeneration (FTLD), the polyglutamine diseases (Huntington’s disease, spinocerebellar ataxia, and dentatorubral–pallidoluysian atrophy). Further studies showed that normal FUS proteins are mainly located in the nucleus, whereas the mutants are primarily found in the cytoplasm [31,32,33]. *FUS* mutations have also been detected in patients with essential tremor (ET) and Parkinson’s disease. For example, a mutation in its exon (c.868C > T) leads to a stop mutation during FUS expression (p.Q290X), resulting in a potential cause of ET [34,35]. Interestingly, FUS usually coaggregates with other FET proteins in the pathologic inclusions of FTLD, while in all cases of ALS with FUS inclusions, the FUS mutant is dominant in the composition of ALS FUS inclusion [36,37]. Further studies showed that normal FUS proteins are mainly located in the nucleus, whereas the mutants are primarily found in the cytoplasm. Most mutations alter the C-terminal nuclear localization signal, resulting in FUS redistribution from the nucleus to the cytoplasm. Excess FUS in the cytoplasm is involved in the formation of stress granules (SGs) during stress, which are membraneless granules composed of mRNAs, ribosome translation initiation factors, and other RNA binding proteins [38,39,40,41]. These granules can be induced by various cellular stresses such as oxidative stress, mitochondrial dysfunction, and viral infection that inhibit translation initiation [42].

FUS has two possible models for cytoplasmic aggregation leading to neurological diseases. One is the gain-of-function model, in which FUS gains a toxic function in the cytoplasm. FUS may sequester important regulators or trigger abnormal signaling pathways to alter cell physiology [43]. The alternative model is that FUS aggregation depletes functional FUS, especially in the dendritic spines of neurons, which can cause instability of mRNA and trigger downstream immune responses such as inflammation, resulting in further neuronal damage [44]. Loss of FUS in the nucleus affects transcription, alternative splicing, and also DNA repair [45]. FUS cytoplasmic aggregation may spread across the anatomical network in a prion-like manner, as observed by biophysical and histological analysis, ultimately leading to neurodegenerative disease [46].

## 2. Domains of the FUS Protein

**Figure 1:**
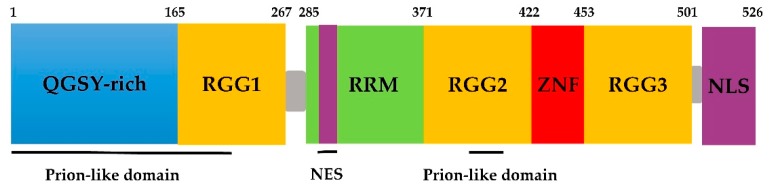
Schematic of fused in sarcoma (FUS) protein domains.

FUS belongs to the FET/TET family, in which proteins share domains similar in both structure and function [47]. FUS contains an N-terminal transcriptional activation domain, which is a glutamine-glycine-serine-tyrosine-rich domain (QGSY-rich domain, amino acids 1–165); three arginine-glycine-glycine repetitive region (RGG, named RGG1–RGG3); an RNA recognition motif (RMM, amino acids 285–371); a Cys2–Cys2 zinc finger (ZnF) motif; a nuclear export signal (NES); and a C-terminus nonclassical nuclear positioning signal (NLS) (Figure 1) [48,49]. In addition, according to bioinformatics analysis, FUS has two prion-like domains (PrLDs) predicted to be amino acids 1–239 and 391–407 [50].

The binding of FUS to RNA may be related to the ZnF motif. The RGG–ZnF–RGG domain is thought to be the major RNA-binding sequence [7,51]. Furthermore, Lerga et al. identified that RNA oligoribonucleotides bind to the FUS protein via the GGUG motif [52]. Survival motor neuron (SMN) proteins are part of a large multiprotein complex that plays a vital role in the biogenesis of small nuclear ribonucleoprotein (snRNP) granules, which results in the fatal childhood motor neuron disease spinal muscular atrophy (SMA) [53]. Sun et al. further demonstrated that the RGG domain in FUS and the Tudor domain in SMN are necessary for the protein–protein interaction [32]. The RGG2 domain recruits FUS to DNA damage sites, which is enhanced by PrLDs [23]. The C-terminal NLS of FUS is recognized by the nuclear input receptor, facilitating the transportation of the protein from the cytoplasm to the nucleus. The majority of mutations discovered in familial ALS is in the NLS. Loss-of-function mutations or deletion in this signaling motif prevents FUS from binding to transport proteins and being transported into the nucleus, and eventually, FUS accumulates in the cytoplasm [54,55].

## 3. FUS Self-Assembly

### 3.1. Prion-Like Domains and Self-Assembly of FUS

Prions are self-replicating proteins with misfolded conformations and can lead to diseases, also known as infectious proteins. A single prion can act as a template to fold soluble proteins containing the same amino acid sequence into the prion conformation, resulting in self-replication [56]. Prions typically form stable amyloid fibers with a hallmark “cross-β” structure [57,58]. Yeast prions such as Sup35, Ure2, and Rnq1 are rich in uncharged polar amino acids (glutamine, asparagine, tyrosine, and serine) and glycines, which are essential for prion-like propagation and amyloid formation, and those areas are referred to as the “prion domain”. Adding this region to other innocuous proteins is sufficient to confer prion behavior [59]. Proteins containing prion-related Q/N-rich domains could induce conformational changes and finally lead to self-assembling, which alters protein functions such as SG formation and synaptic translation [60]. The most neurodegenerative diseases share similar cellular and biochemical mechanisms, which is the accumulation of misfolded proteins in brains. Thus, numerous reports suggest that aggregation-prone RNA-/DNA-binding proteins with PrLDs cause neurodegenerative disease by seed formation in a prion-like manner [41,61]. In other words, misfolded proteins act as seeds of aggregation, folding their primary subtypes and converting them into pathological aggregates, which ultimately generate neurological diseases through further recruitment and transformation [62]. A typical example of a pathogenic PrLD is that the trinucleotide is repeatedly amplified in the gene encoding ataxin 1 (ATXN1) in PrLDs and promotes aggregation of ATXN1, leading to polyglutamine protein production and, ultimately, to spinocerebellar ataxia type 1 [61,63,64,65].

The experimental results from the interactions between FUS and polyglutamine inclusions and the predictions suggested that the FUS prion-like domains are located in the N-terminal QGSY domain and are part of the first RGG domain (amino acids 1–239), with an additional region in the RGG3 domain (amino acids 391–405) [66,67]. By using a prediction algorithm based on a yeast prion domain to score 27,879 human proteins, FUS ranked 15th for its prion-like property and 1st in RNA binding proteins [45]. Prion-like domains in RNA-binding proteins are essential for neuronal proteins to enter ribonucleoprotein granules, forming a functional assembling state, which also drives pathological protein aggregation in neurons. It can therefore be speculated that the prion-like domain in FUS is essential for its aggregation in neurons [68]. It was reported that the PrLD of FUS can be assembled into amyloid-like fibrils in a cell-free system and the morphology of aggregates is similar to the general pathogenic amyloid fibers. Reports showed that the process is reversible and highly regulated. Amyloid fibrils formed by FUS are susceptible to depolymerization due to a variety of factors, including FUS concentration, DNA or RNA levels, and SYGQ-rich domain phosphorylation [50,69,70,71,72,73,74].

Phase separation is a physical process in which a supersaturated solution of components spontaneously separates into two phases with different densities, both stable and coexisting [75]. This phenomenon is common in polymer chemistry and has recently been found in biomacromolecules [76]. For example, FUS undergoes liquid–liquid phase separation (LLPS) both in vivo and in vitro at physiological concentrations [77]. It is progressively recognized that phase separation controls the formation of membrane-free organelles, regulating biological functions and activities [78]. FUS is rapidly recruited to the site of DNA damage, then phase separation allows FUS to locally form droplet-like compartments, increasing the concentration of FUS and other substances and possibly promoting DNA repair [79,80].

Studies have shown that FUS has three forms of existence—dispersion, droplets, and hydrogels—and the reversible dynamic phase transition is driven by the low-complexity (LC) domain at the N-terminus of FUS [81]. According to the results of Patel et al., FUS closed compartments have two states of droplets and hydrogels which are reversible and dynamic [82]. Their experiments showed that increasing the concentration of FUS in vivo or in vitro causes its transformation from liquid to an aggregated state. Under stress conditions, FUS rapidly passes through the nucleus to the cytoplasm, then forms SGs [83]. SGs act as condensation sites, where the FUS concentration is locally increased and eventually may aggregate [3].

**Figure 2:**
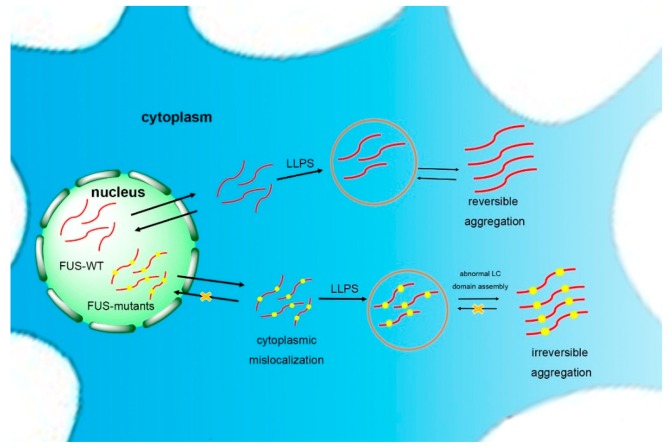
Self-assembling model of FUS. FUS can phase separate to form granules and reversible aggregates. Once the self-assembly of the low-complexity (LC) domain goes beyond normality, usually caused by mutations in neurodegenerative diseases such as amyotrophic lateral sclerosis (ALS), FUS will form irreversible aggregation, possibly via liquid–liquid phase separation (LLPS).

The basis of the reversible phase separation of FUS is the self-assembly of the LC domain [69]. Once the self-assembly of the LC domain goes beyond normality, usually caused by mutations in ALS, FUS is driven to form a poorly soluble and stable hydrogel (Figure 2) [45,84]. Murakami et al. demonstrated that pathogenic FUS mutations reduce the ability for reversible phase transitions. More specifically, the liquid-to-solid transition is accelerated by FUS mutants, indicating that FUS phase transitions are closely related to disease. In addition, the repeated cycles of gelation and degelation of FUS promote it to form an irreversible hydrogel [85]. The droplets and hydrogels formed by the LC domains spontaneously mature into fibrous solid aggregates as the cell ages and the ability of the quality control mechanism diminishes [82]. However, the rest of the FUS domains will reduce the tendency of FUS assembly to hinder the formation of high-grade assemblies of the full-length protein. Meanwhile, specific cellular proteins, such as the receptors transportin (TNPO1) and karyopherin-b2 (Kapb2), inhibit the solidification of FUS [86,87]. Structural analysis has shown that the LC domain noncovalently aggregates into morphologically uniform amyloid fibrils in FUS protein hydrogels [75], with a structure similar to FUS aggregates in the cytoplasm of spinal motor neurons of FUS mutant patients (with amyloid protein-like prominent filamentous cross-β structure) [88].

### 3.2. Amyloid Core in LC Domains of FUS Regulates Its Self-Assembly

Recent solid-state nuclear magnetic resonance (NMR) studies have indicated a structurally ordered amyloid core in the LC domain of FUS, also known as the prion-like domain. At the same time, it was discovered that the residues 39–95 constitute the structurally ordered core of the prion-like domain fibers in combination with the computational assistance method based on the MCASSIGN algorithm [89,90]. The droplets and hydrogels formed by FUS are stabilized by hydrogen bonds between the antiparallel β-sheet conformations [90]. The hallmark structure of pathogenic amyloid fibers is an in-register parallel cross-β structure in which the β-strands are perpendicular to the fiber axis, generally formed from 2 to 9 repetitive residues [91]. β-strands, irregular crimps, and loops constitute the backbone conformation of the fibers and the β-sheet layer interacts by van der Waals forces [92,93]. Sayawa et al. named this structure a “stereo zipper” that is highly stable and generally resistant to high temperatures, proteases, and detergents [94]. The FUS-LC fibrils have a single cross-β unit, with all molecules having the same structural environments and conformations and a cross-β-core-aligned parallel intermolecular alignment.

The overall bone architecture of residues 43–95 and the orientation of the side chains of residues 44–52 and 65–95 have been determined, while residues 55–62 form a dynamic loop. The fragments of the β-strands forming the cross-β contain residues 44–46, 52–54, 62–64, 67–70, 85–90, and 93–95, determined from the characteristic arrangement of the backbone carbonyl and the fiber growth axis [90]. This also indicates that many of the structurally ordered cores of the FUS-LC fibers do not participate in the formation of hydrogen bonds, which makes the cross-β structure more stable [90]. Probably because of the more rational arrangements, residues 39–95 dynamically convert into stable cross-β structures, driven by entropy cost or desolvation energy [95]. From the structural model, it is known that S84 forms a hydrogen bond network with Y75 and T78, and the stability of the loop between 74 and 87 is related to this hydrogen bond network. Phosphorylation sites S84 and S87 are located between the loops 74 and 87; therefore, the disruption of the loop conformation may be critical to the formation of FUS-LC fibers [90]. In addition, the folding of residues 44–50 against residues 64–80 is stabilized via the interaction of S48 with Q69 and T71 and the interaction of T45 and T47 with S77. The folding of residues 69–73 to 90–95 may be stabilized by the interaction of S70, Q73, S90, Q93, and S95 with each other. Y50 and Y66 may participate in π-stacking interactions (attractive, noncovalent interactions between aromatic rings) which help in stabilizing the core structure [90]. In conclusion, hydrogen bonding and electrostatic dipole–dipole interactions between polar side chains may improve the stability of the PrLDs of the FUS structure. The prion-like domain fibers of FUS lack pure hydrophobic side chains, which are conducive to disassembly [74].

Liu et al. found that 37SYSGYS42 and 54SYSSYG59 in the amyloid core region 39–95 can form reversible fibers that are sensitive to temperature and phosphorylation, termed the reversible amyloid nucleus [96]. Reversible amyloid cores (RACs) are assembled at low temperatures and disassembled as the temperature increases. The absence of either 37SYSGYS42 (RAC1) or 54SYSSYG59 (RAC2) significantly reduces the ability of self-assembling for FUS-LC; thus, it is considered the core area for FUS self-assembly [96]. Using microelectronics and X-ray diffraction techniques, Liu et al. determined that RAC1 is not a fiber spine of β-strands but a coil arranged along the fiber axis with Gly40 as a kink point. The hydroxyl group of the same layer of Tyr38 forms a hydrogen bond with the carbonyl group of Tyr41 to block the coil, and the adjacent sheets of Tyr38 and Ser42 also form hydrogen bonds, which facilitates the formation of a hydrogen bond network [96]. When the formation of these hydrogen bonds is disrupted, such as by Ser42 phosphorylation, the RAC1 fiber disassembles [24]. The π-stacking of Tyr38 enhances the interaction of adjacent layers. The RAC1 fibril spine uses polar residues to form the interface between the mating layers, which is different from the hydrophobic interface composed of nonpolar residues of pathogenic fibers [97]. Additionally, the interaction between the pathogenic fibrous sheets also has hydrogen bonds and van der Waals forces, resulting in a relatively stable fiber structure [96,98]. Unlike RAC1, in the crystal structure, the RAC2 fiber contains a cross-β structure, the marker of the pathogenic fiber. However, in the RAC2 fibril spine, water molecules, together with the hydroxyl groups of Tyr58, form a hydrogen bond, so that the distance between the sheets increases to 13 Å, while the spacing of the layers of normal pathogenic fibers is ~10 Å. The interlayer bonding is not tight and the fiber is remarkably unstable [94,96]. The unique structure of the fibers formed by RAC1 and RAC2 makes them reversible, facilitating FUS reversible self-assembly [69,96].

## 4. Regulation Factors of FUS Self-Assembly

The physiological function of FUS is performed while in a droplet state. In vitro, FUS droplets are amorphous, and it is speculated that, in vivo, FUS may have no ordered structure, and the oligomers formed may be gradually decomposed [99,100]. Currently, the confirmed mechanism for driving droplet formation is through its prion-like LC domains [82,101]. It can therefore be hypothesized that increasing the interaction of the LC domains accelerates the formation of aggregates. Conversely, weakening the interaction of the LC domain can be detrimental to liquid–liquid phase separation [77,101]. Irreversible FUS aggregates selectively capture ribonucleoproteins, thereby disrupting RNP granule function and impeding the synthesis of new proteins at the axon terminals of neuronal cells [85,102]. DNA-PK phosphorylates the LC domain of FUS [27]. Tycko et al. identified 14 phosphorylation sites, including 30, 42, 54, 61, 84, 87, 112, 117, 131, and 142 as well as Thr residues 7, 11, 19, and 68. In the fibril core formation segment, the phosphorylation of Ser and Thr residues disrupts the interaction of the cross-β, inhibiting hydrogel binding and droplet formation [69]. RAC1 does not form amyloid fibrils when Ser42 is phosphorylated, and S42D mutation significantly inhibits phase separation of FUS-LC [96].

Numerous studies have shown that the regions of FUS other than the LC domains, especially the C-terminal domain, are important in the phase transition behavior of FUS. Qamar et al. demonstrated that the synergistic cation–π interaction between the tyrosine of the LC domain and arginine of the structured C-terminal is related to phase separation [103]. The electrons of the tyrosine benzene ring form a cation–π interaction with the protons in the guanidino moiety of arginine, resulting in a local high concentration of the LC domains. Methylation of arginine is a common post-translational modification of RNA-binding proteins, and the C-terminal arginine rich in FUS is modified by methyltransferase with asymmetric dimethyl groups [104]. This changes the local hydrophobicity and hydrogen bonding of arginine, thereby affecting the cation–π interaction [105]. The degree of methylation of arginine in the C-terminus regulates FUS assembly. Arginine hypomethylation at the C-terminus domain greatly accelerates phase separation and gelation, allowing FUS to form a stable hydrogel containing β-sheets. This is consistent with hypomethylation in FUS-related patients with frontotemporal degeneration [75,106]. Experiments indicated that unmethylated FUS accounts for more than 1% of the total amount of FUS, which can induce the dispersed FUS to assemble into droplets and, in turn, become a stable fibrous gel [103].

The nuclear import receptor TNPO1 and Kapb2 regulate FUS phase separation by binding to FUS via its C-terminal domain [54]. They exist in the axonal terminal compartment of neurons, mediating nuclear import of FUS. TNPO1 acts as a molecular chaperone to reduce the tendency of FUS to form stress granules, suppressing FUS phase separation and liquid–solid phase transition. TNPO1 plays a central role in the FUS quality control mechanism. The highly stable combination of Kapb2 and FUS NLS weakens and dynamically interacts with the self-assembly-related regions, hindering the self-assembly of FUS. In vivo, Kapb2 dissociates FUS and RNA, regulating cytoplasmic RNA particles [107]. In vitro, Kapb2 dissolves the FUS phase separated liquid and aberrant fibrous hydrogels [108].

It is currently known that over 50 mutations/deletions in *FUS* are associated with ALS, accounting for about 5% of familial cases (FALS) and 0.7–1.8% of sporadic cases (SALS) [36]. Mutations in *FUS* mainly occur in exons 3–6 and exons 12–15. According to statistics, approximately two-thirds of *FUS* mutations are in exons 12–15, which encode ZnF motif, RGG2 and RGG3, and nuclear localization signaling domains. In particular, more than 50% of ALS-associated FUS mutations were found in the C-terminal NLS domain. Exons 3–6 encode the QGSY-rich and RGG1 regions, and about one-third of the mutations are in them [30,88,109]. The known mutations of *FUS* are shown in Table 1. A series of mutations regulate the self-assembling of FUS. NLS mutations, on the one hand, can reduce the nuclear import, increasing the concentration of cytoplasmic FUS and promoting the formation of a more durable and mature fiber structure; on the other hand, they can change the kinetics of phase transition [82,85]. It has been shown that the NLS mutant of FUS reduces the binding of TNPO1, such as P525L and R495X, affecting the nuclear import of FUS [75]. The NLS mutations of FUS cause the compulsive accumulation of FUS in the cytoplasm, resulting in aggregates that may produce toxic effects and trigger neuronal degeneration [109]. In 2011, Suzuki and colleagues constructed a series of FUS-deleted domains and explored the SG formation of them. The results showed that C-terminal deletion formed a large number of stress granules, indicating that the C-terminus is important in stress granule formation. Later studies showed that mutation R514S or P525L formed SGs in HeLa cells, and double mutations of R514S and P525L or triple mutations of R514S, R521C, and P525L promoted the formation of stress granules [82]. Mutations in PrLDs accelerate the conversion of liquid to hydrogels. The G156E mutant tended to increase the aggregation of FUS in a manner of seed aggregation, causing rapid progression of ALS by using aggregated protein fibrils as a structural template for promoting nonaggregated protein fibrosis [85,110].

Unlike the mechanism by which the mutated FUS locates in the cytoplasm, the FALS-associated *FUS* mutation enhances the interaction between FUS and SMN and reduces its levels at the normal expression sites, thereby impairing motor neurons [53]. Primarily mutant FUS impairs neuronal survival and causes defects in dendritic growth and synaptic connectivity by interfering with the ability of FUS in DNA damage repair and RNA splicing, leading to neurological disorders [12]. Mutations of FUS in the glycine-rich domain (amino acids 156–262) and the C-terminal domain (amino acids 450–526) affect its interaction with the chromatin remodeling factor histone deacetylase 1, which plays a fundamental role in DNA repair and genomic stability maintenance via interaction with FUS. Experiments by Qiu et al. showed that there is no wild-type FUS and interaction in FUS-R521C transgenic mice due to the mutated FUS-R521C protein in those mice, with HDAC1 forming a more stable complex with the wild-type FUS protein, implying that the mutated FUS acquired abnormal functional properties [4]. The study of Nishimoto et al. indicated that FUS affects the integrity of paraspeckles (subnuclear structures that regulate gene expression by nuclear retention of RNA) by interacting with a long noncoding RNA nuclear-enriched abundant transcript (NEAT1), and mutations of FUS may disrupt integrity and maintenance of paraspeckles [35,149]. In the same year, cytoplasmic aggregates of P45NRB/NONO, which are core proteins of paraspeckles, were found in spinal motoneurons in transgenic mice expressing truncated FUS mutant proteins (amino acids 1–359) [150]. This verified the FUS-mutation-disrupting integrity of paraspeckles.

## 5. Expectations

Most neurodegenerative diseases are associated with the misfolding of proteins. A growing number of studies have shown that FUS mutations in prion-like domains mediate the aggregation of FUS, which is an important reason for the development and progression of most neurodegenerative diseases. The mechanism of action of prion-associated regions of FUS needs to be further investigated, such as under what conditions the pathological aggregation of FUS occurs and whether they can play a role in disease progression by allowing the cell–cell metastasis of pathological protein aggregates. Although emergency particle assembly and prion proliferation has been confirmed, whether FUS mutations associated with neurodegenerative diseases cause normal function loss, a gain of toxic properties of FUS aggregates, or a combination of the both requires clarification and further studies. In addition, based on known mechanisms, the research of small-molecule-targeting inhibitors of FUS is also a topic of particular interest regarding finding a cure for related diseases. We are optimistic that with the deepening of FUS research, this field will make a major breakthrough.

## Figures and Tables

**Table 1:** Summarizes of the mutations in the *FUS* gene. ALS: amyotrophic lateral sclerosis; FTLD, frontotemporal lobar degeneration; ET, essential tremor.

NO.	Exon	Domain/Region	Nucleotide Change	Amino Acid Mutation	ALS	References
1	3	PrLDs	c.52 C > T	p.P18S	ALS	[111]
2	3	PrLDs	c.170_172delCTT	p.S57del	ALS	[112]
3	4	PrLDs	c.287_291delCCTACinsAT	p.S96del	ALS	[113]
4	4	PrLDs	c.344 G > A	p.S115N	ALS	[114]
5	5	PrLDs	c.430_447delGG	p.G144Y149del	ALS	[111,115]
6	5	PrLDs	c.467 G > A	p.G156E	ALS	[116]
7	6	PrLDs	c.518_523del GAGGTG	p.G174_G175del	AlS/FTLD	[30,113]
8	6	RGG1	c.616 G > A	p.G206S	AlS/FTLD	[113]
9	6	RGG1	c.646 C > T	p.R216C	ALS	[117]
10	6	RGG1	c.674 G > T	p.G225V	ALS	[117]
11	6	RGG1	c.681_684delGGC	p.G230delG	ALS	[118]
12	6	RGG1	c.688 G > T	p.G230C	ALS	[117]
13	6	RGG1	c.700 C > T	p.R234C	ALS	[117]
14	6	RGG1	c.701 G > T	p.R234L	ALS	[116]
15	6	RGG1	c.730 C > T	p.R244C	ALS	[30]
16	6	RGG1	c.734 G > T	p.G245V	ALS	[119]
17	9	RRM	c.868 C > T	p.Q290X	ET	[120]
18	11	RGG2	c.1129 C > T	p.R377W	ET	[121]
19	12	PrLDs	c.1176 G > A	p.M392I	ET	[122]
20	12	PrLDs	c.1196 G > T	p.G399V	ALS	[118]
21	12	RGG2	c.1204 1232 delinsGGAGGTGGAGG	p.S402P	ALS	[123]
22	13	ZNF	c.1292 C > T	p.P431L	ET	[124]
23	13	RGG3	c.1385 C > T	p.S462F	ALS	[125]
24	13	RGG3	c.1392 G > T	M464I	ALS	[126]
25	14	RGG3	c.1394_1541del	p.G466VfsX14	ALS	[123]
26	14	RGG3	c.1420_1421insGT	p.G472VfsX57	ALS	[127]
27	14	RGG3	c.1449_1488del	p.Y485AfsX514	ALS	[113]
28	14	RGG3	c.1456_1457delGG	p.G486PfsX30	ALS	[128]
29	14	RGG3	c.1459 C > T	p.R487C	ALS	[114]
30	14	RGG3	c.1464 C > T	p.G488G	ALS	[119]
31	14	RGG3	c.1475delG	p.G492EfsX527	ALS	[129]
32	14	RGG3	c.1483delC	p.R495EfsX527	ALS	[113]
33	14	RGG3	c.1483 C > T	p.R495X	ALS	[39,130,131]
34	14	RGG3	c.1484delG	p.R495QfsX527	ALS	[132]
35	14	RGG3	c.1485delA	p.G497AfsX527	ALS	[113]
36	14	RGG3	c.1489_1490dupGG	p.R498AfsX32	ALS	[128]
37	14	-	c.1506dupA	p.R502fsX15	ALS	[111]
38	14	-	c.1507_1508delAG	p.G503WfsX12	ALS	[118]
39	14	-	c.1509_1510delAG	p.G504WfsX12	ALS	[130]
40	14	-	c.1520 G > A	p.G507D	ALS	[117,133,134]
41	14	NLS	c.1526 G > A	p.G509D	ALS	[119]
42	14	NLS	c.1527_1528insTGCC	p.K510WfsX517	ALS	[113]
43	14	NLS	c.1528 A > G	p.K510E	ALS	[135,136]
44	14	NLS	c.1529 A > G	p.K510R	ALS	[131]
45	14	NLS	c.1537 T > C	p.S513P	ALS	[135]
46	14	NLS	c.1540 A > G	p.R514G	ALS	[88]
47	14	NLS	c.1542 G > C	p.R514S	ALS	[30,137,138,139,140]
48	15	NLS	c.1543 G > T	p.G515C	ALS	[30]
49	15	NLS	c.1544delG	p.G515VfsX14	ALS	[128]
50	15	NLS	c.1547 A > T	p.E516V	ALS	[140]
51	15	NLS	c.1549 C > G	p.H517D	ALS	[141]
52	15	NLS	c.1550 A > C	p.H517P	ALS	[135]
53	15	NLS	c.1551 C > G	p.H517Q	ALS	[30]
54	15	NLS	c.1552 A > G	p.R518G	ALS	[104]
55	15	NLS	c.1553 G > A	p.R518K	ALS	[30]
56	15	NLS	c.1554_1557delACAG	p.R518del	ALS	[142]
57	15	NLS	c.1555 C > T	p.Q519X	ALS	[112]
58	15	NLS	c.1561C > T	p.R521C	ALS	[112,143,144]
59	15	NLS	c.1561 C > G	p.R521G	ALS	[115,133]
60	15	NLS	c.1561 C > A	p.R521S	ALS	[139]
61	15	NLS	c.1562 G > A	p.R521H	ALS	[112,139,145,146]
62	15	NLS	c.1562 G > T	p.R521L	ALS	[113,139,147]
63	15	NLS	c.1564 C > T	p.R522G	ALS	[30]
64	15	NLS	c.1570 A > T	p.R524W	ALS	[134]
65	15	NLS	c.1571 G > C	p.R524T	ALS	[30]
66	15	NLS	c.1572 G > C	p.R524S	ALS	[30,113]
67	15	NLS	c.1574 C > T	p.525L	ALS	[142]
68	15	NLS	c.1574 C > G	p.525R	ALS	[142]
69	15	NLS	c.1575_1576insTAT	p.P525_Y526ins	ALS	[148]
70	15	NLS	c.1581delA	p.X527YextX	ALS	[118]
